# Effect of the Crack Tip Bifurcation on the Plasticity-Induced Fatigue Propagation in Metallic Materials

**DOI:** 10.3390/ma14123385

**Published:** 2021-06-18

**Authors:** Jesús Toribio, Beatriz González, Juan-Carlos Matos

**Affiliations:** Fracture & Structural Integrity Research Group (FSIRG), Campus Viriato, University of Salamanca (USAL), E.P.S., Avda. Requejo 33, 49022 Zamora, Spain; bgonzalez@usal.es (B.G.); jcmatos@usal.es (J.-C.M.)

**Keywords:** crack tip bifurcation, finite element method, plasticity-induced fatigue crack propagation, retardation phenomenon

## Abstract

This article deals with the influence of the crack path branching (at the micro level) on the plasticity-induced fatigue crack growth. With regard to this, a modeling by means of the finite element method was performed considering a cracked panel subjected to tension with different symmetric and asymmetric configurations of the bifurcated crack tip. The results show the appearance of a retardation effect in the growth rate of the bifurcated crack in relation to the growth rate of the fully straight crack in different cases studied, namely: (i) if the two branches of the bifurcation have different initial projected length, the propagation rate is greater at the crack tip corresponding to the long-branch than that of the short-branch, and the long-branch growth rate increases with the decrease of the initial branch angle and of the initial projected short-branch length and with the increase of the intensity of fatigue; (ii) if the two branches of the bifurcation have identical initial projected length, the retardation effect depends on the initial distance between the two bifurcated crack tips, the growth rate going up with the decrease of such a distance and with the increase of the fatigue intensity.

## 1. Introduction

In steel, cracks propagating by fatigue in (macroscopic) mode I show in their path (at the microscopic level) multiple deflections and bifurcations, changes in the crack opening, etc., phenomena that will produce micro-roughness on the fracture surface. Additionally, the application of tensile overloads produces deflection and bifurcation of the crack [1], overload-induced fatigue crack branching being a well-known mechanism of retardation (even arrest) of the crack growth that can quantitatively explain these effects even when specific arguments cannot be used in the matter of crack closure induced by plasticity [2].

With regard to the role of microstructure in fatigue crack propagation (and very particularly on the topics of *fatigue crack paths* and *crack bifurcation*), the analysis of fatigue crack growth behavior under cyclic loading with constant stress intensity factor (SIF) range in the Paris regime for a steel with coarse grain shows that the crack tip stress shielding induced by crack deflection and crack branching or bifurcation (occasioned by crystal orientation) mainly contributed to fatigue crack growth resistance in this material, i.e., the tortuosity of the crack path induces a better performance regarding fatigue crack propagation [3].

In the matter of the influence on fatigue crack propagation (*fatigue crack paths* and *crack bifurcation* issues) of microstructural orientation and densification produced by the manufacturing of cold drawn pearlitic steel wires by progressive (multi-step) cold drawing, it was seen [4,5] that the afore-said microstructural arrangement and peculiarities (orientation and densification) produce a fatigue crack path with a greater surface micro-roughness (by branching, bifurcations and local deflections) that must be considered when evaluating the fatigue crack propagation rate, as described elsewhere [4,5], i.e., again the micro-roughness and tortuosity of the fatigue crack path induce in the material a greater resistance to fatigue crack growth, cf. [4,5].

With regard to crack bifurcation, outer and inner branches produce an accentuated trend to growth in the direction of the original crack [6]. Additionally, the SIF of a branched crack can be considerably smaller than that of a straight crack with identical projected length [7]. For symmetric crack branching, the SIF value decreases with the branch length (if it is small compared to the total crack length) [8] and, during its propagation in the fracture process, a particular stable branch angle can be observed for each state of biaxial tension, as it increases with the lateral biaxial tension stress [9].

Computations performed for cases of asymmetric branching (with two branches of different length) indicate a strong effect of this asymmetry on the SIF of both branches; the SIF of the long-branch will be considerably greater than that of the short-branch, even at fairly small differences of branch length [2]. In a quasi-dynamic analysis, energy balance considerations are used to study the propagation of two branches of distinct lengths. It is seen that at low velocities the short-branch be quickly arrested, while at very high velocities the two branches will continue to grow with nearly the same velocity (increasing the energy dissipation very quickly) [6].

Plasticity-induced cyclic crack growth modeling has provided interesting results related to the variation of the crack propagation rate with the load range and load ratio, and with single over- and under-load cycles [10]. This numerical approach has also been used to analyze the appearance, or not, of the plasticity-induced crack closure phenomenon during crack cyclic propagation [10,11,12,13,14], to calculate the Paris parameters (characteristics of the Paris regime) of different steels with very similar results to experimental values [15], and to characterize the retardation in the crack propagate rate produced by the micro-deflection of the crack tip, it being greater as the kinked crack tip angle increases [16].

An iterative method to simulate the propagation of fatigue cracks in 3D (based on the plastic activity around the crack tip) in a BCC single crystal with different sliding systems (under cyclic loading) provides non-regular crack shapes, such as the paths of zigzag cracks [17].

Fatigue involves both monotonic and cyclic plasticity, resulting in two-load or stress parameter requirement, independent of any hypothetical crack closure that could be assumed to be present [18]. However, using a numerical prediction method based on cumulative plastic strain at the crack tip, it has been concluded that maximum SIF has no effect on cyclic plastic deformation at the crack tip itself, so the approaches assuming a two-parameter driving force based on maximum SIF and SIF range are implicitly proposing other crack tip damage mechanisms [19].

From experimental data obtained in fatigue crack growth tests under quasi-constant load conditions (SIF range and maximum SIF) in steel and aluminum specimens, it has been concluded that Elber’s effective SIF range was not the actual fatigue crack growth driving force for the analyzed tests [20]. Thus, there seems to be no common agreement among the researchers regarding whether a single or two-parameter driving force is more suitable for fatigue crack growth analyzes [21].

The main aim of this paper is to study the retardation in plasticity-induced fatigue crack growth rate due to the bifurcation of the crack tip, the key variables analyzed being the geometrical configuration of the crack tip (different projected length and equal angle for the two branches, equal projected length and different angle for the two branches and symmetric case, i.e., equal projected length and equal angle for the two branches), the initial crack tip geometry (branch projected length and angle measured in relation to the direction of the macroscopic crack), and the intensity of the fatigue (characterized by the SIF range).

## 2. Numerical Procedure

For the study of fatigue crack propagation by plastic crack advance, a numerical simulation by using the finite element method (FEM) under small scale yielding (SSY) was performed by means of the commercial MSC.Marc software (a powerful nonlinear finite element code).

Material was elastic/perfectly-plastic steel with the properties correspond to a typical low-medium strength steel: Young’s modulus *E* = 200 GPa, Poisson coefficient ν = 0.3 and yield strength *σ*_Y_ = 300 MPa. In this modeling, the von Mises yield criterion, large strains and large geometry changes were used with an updated Lagrangian formulation.

**Figure 1:**
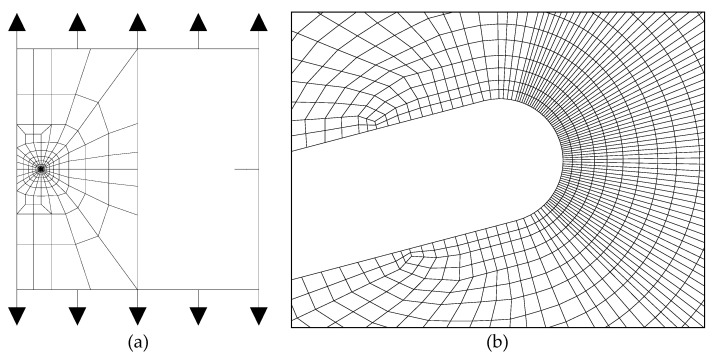
Finite element mesh: (**a**) general view on the full plate; (**b**) crack tip detail.

The plasticity-induced fatigue propagation was analyzed in a symmetric double-edge-cracked plate subjected to remote tension cyclic loading (Figure 1a). The initial crack (undeformed) was a parallel-flanks sharp notch with bifurcated crack tip and circular geometry at its ends (Figure 1b).

It was only necessary to simulate half of the plate (with the appropriate boundary conditions) due to the symmetry of the problem (Figure 1a). The mesh design was based on the one described in [10,11,12], modifying it according to the particular geometry of the problem to account for bifurcated cracks and deflection angles.

The computational study was led under plane strain conditions (the more realistic and conservative in the matter of fracture mechanics analyses and evaluations) and using the well-known four-node isoparametric quadrilateral elements to build the finite element mesh.

Mesh refinement was considered to be adequate after successful previous research [10,11,12] and additional convergence analyses carried out in the framework of the present numerical analysis with deflected and bifurcated cracks, showing that even with less refined meshes the numerical results converged to a unique solution.

**Figure 2:**
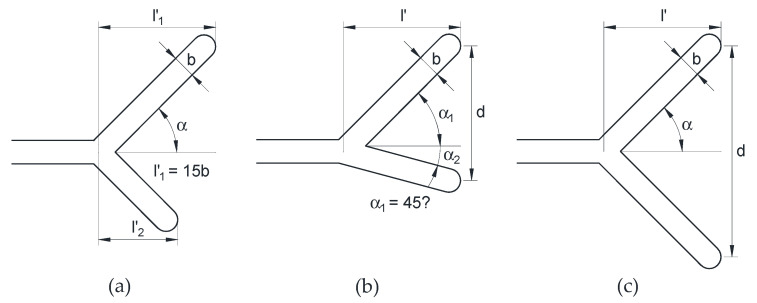
Initial geometry for the three bifurcated crack configurations: (**a**) different projected branch lengths; (**b**) different branch angles; (**c**) symmetric case.

Three initial crack configurations (undeformed) were studied in the present paper (in addition to the totally straight crack or reference configuration) in which the crack tip presented different particularities at the microscopic level: bifurcated crack tip with different projected lengths and equal angle in the two branches (Figure 2a); bifurcated crack tip with equal projected length and different angles in the two branches (Figure 2b); symmetric bifurcated crack tip, i.e., equal projected length and equal angle in the two branches (Figure 2c).

The parameters characterizing the initial geometry of the bifurcated crack tip (Figure 2) are as follows: *α*, angle of deflection of each branch in relation to the macroscopic crack; *l*’, projected length of each branch in the direction of the macroscopic length of the crack; *d*, distance between the crack tip points of each branch (corresponding to the greater depth in the macroscopic direction of the crack). When the two branches of a bifurcation have different values of the parameter *α* or *l*’, a subscript indicating the branch number has been attached to its symbol.

The plate had the same height as the width and the projected length of the crack was a fifth part of the plate half-width and 15,000 times the crack width. The crack width *b* was 5 µm, so that the notch is sharp enough that it can be considered as a crack and the problem control is in the SIF (according to fracture mechanics theory). For the crack with bifurcated tip, the other dimensions characterizing the initial geometry are shown in Table 1 for the different configurations.

## 3. Numerical Results

### 3.1. Straight Crack

**Figure 3:**
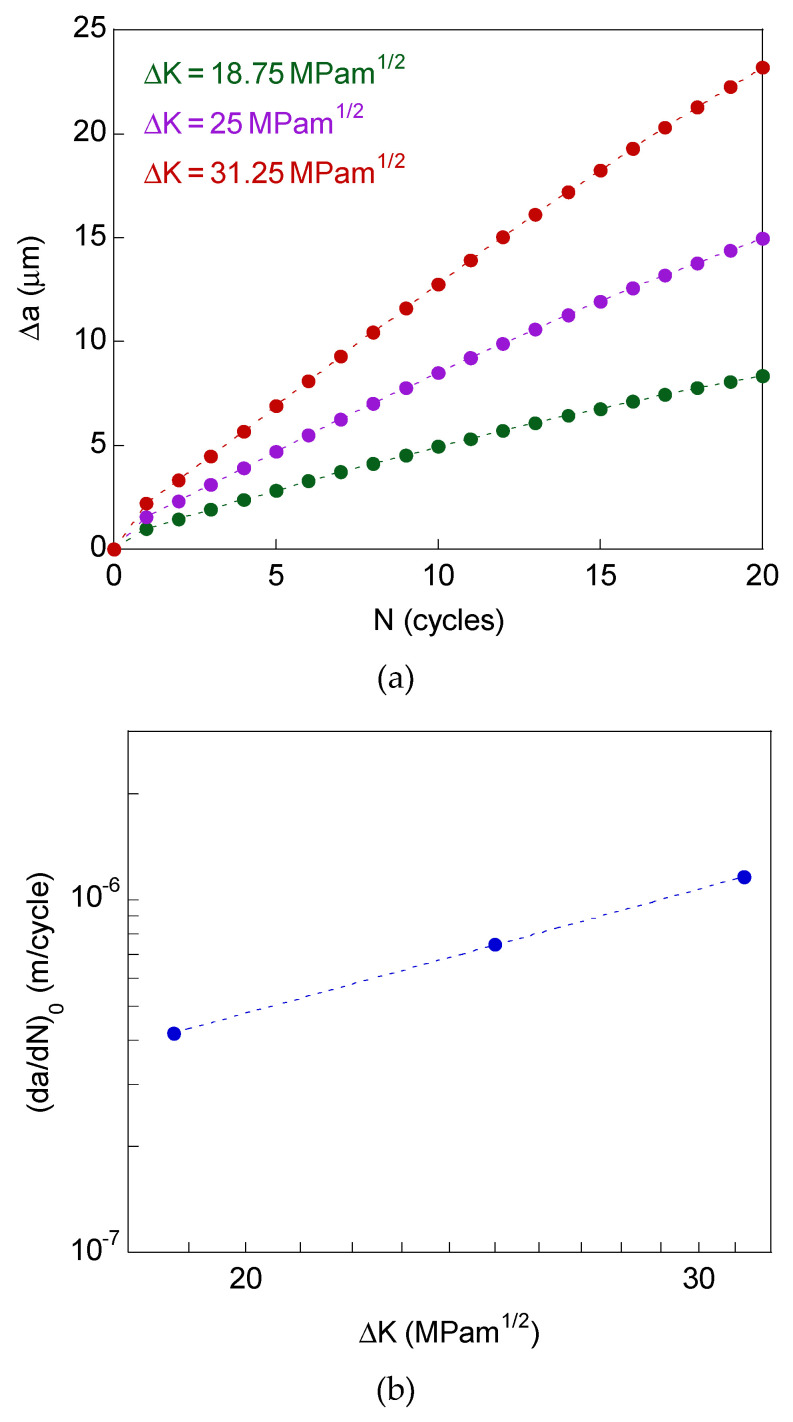
Plastic fatigue propagation for straight crack tip: (**a**) crack growth vs. number of cycles; (**b**) crack growth rate vs. SIF range (Paris curve).

For the modeling with the fully straight crack (without micro-bifurcations), Figure 3a plots the crack growth vs. number of cycles (Δ*a*–*N* *curves*) of the steel for the three SIF ranges associated to the Paris regime (with *R*-ratio equal to 0), namely, Δ*K* = 18.75, 25, and 31.25 MPam^1/2^. These plots, associated with 20 loading cycles, are approximately straight.

Figure 3b shows the cyclic crack growth plot, i.e., plastic fatigue crack growth rate vs. SIF range (d*a*/d*N*-Δ*K curve*), in bilogarithmic scale. The propagation rate by plastic flow was calculated as the average value measured after 20 loading cycles. The fit of these data allows one to obtain the Paris parameters of the material. In this numerical modeling, the crack extends by cyclic blunting and re-sharpening (in agreement with the Laird-Smith mechanism), transferring material from the crack tip towards the crack flanks, while the plasticity-induced crack closure has never been observed [10,11,12].

### 3.2. Bifurcated Crack Tip

In this study about the effect that the micro-bifurcation of the crack tip produces on fatigue advance, it is observed how the crack tends to propagate in mode I when it is subjected to remote mode I (opening) tensile loading, a retardation effect appearing in the matter of crack propagation rate.

The afore-said phenomenon is characterized throughout this paper by the so-called retardation factor (d*a*/d*N*)/(d*a*/d*N*)_0_, where (d*a*/d*N*) is the growth rate when the crack has its bifurcated tip and (d*a*/d*N*)_0_ is the growth rate when the crack is fully straight (reference situation).

Both crack growth rates are obtained as the average value after applying 20 cycles loading, taking into account the crack end point farthest from the crack mouth in the crack macroscopic direction. A small retardation factor implies a smaller crack growth rate and, therefore, a greater retardation effect.

**Figure 4:**
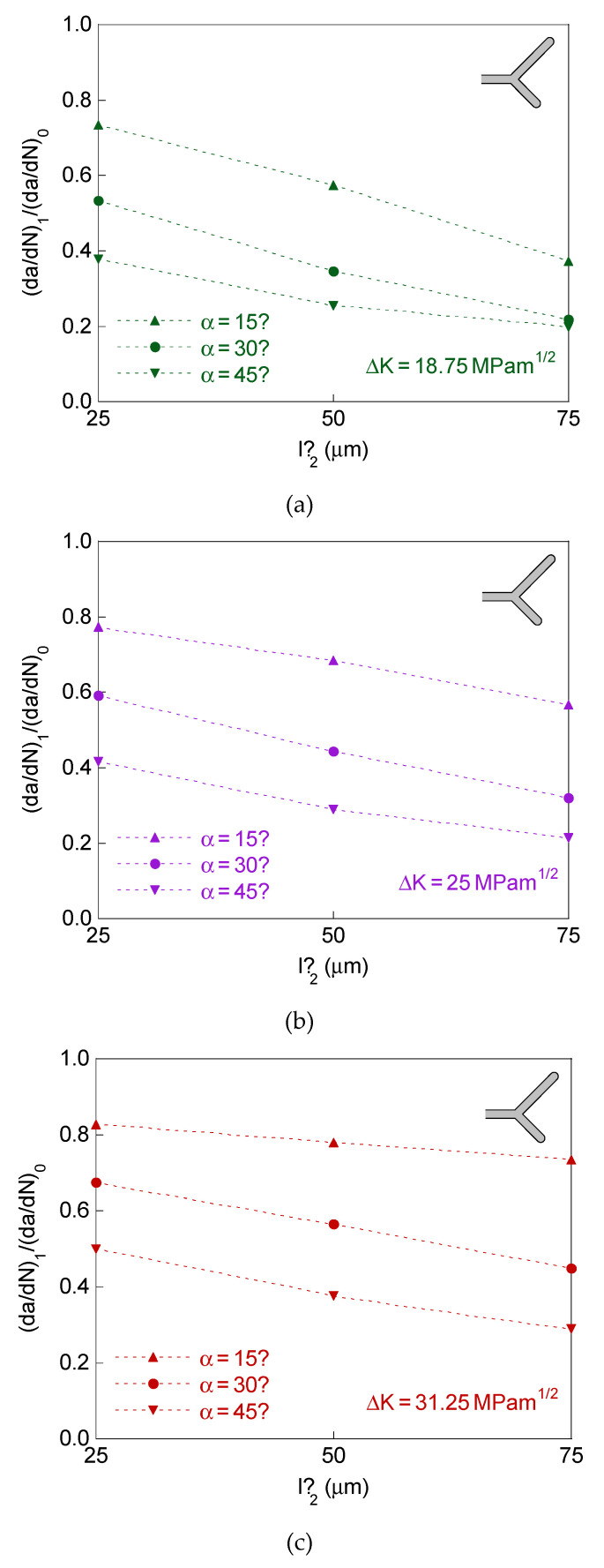
Retardation factor for bifurcated crack tip with different initial projected length and equal initial angle in the two branches: (**a**) Δ*K* = 18.75 MPam^1/2^; (**b**) Δ*K* = 25 MPam^1/2^; (**c**) Δ*K* = 31.25 MPam^1/2^.

**Figure 5:**
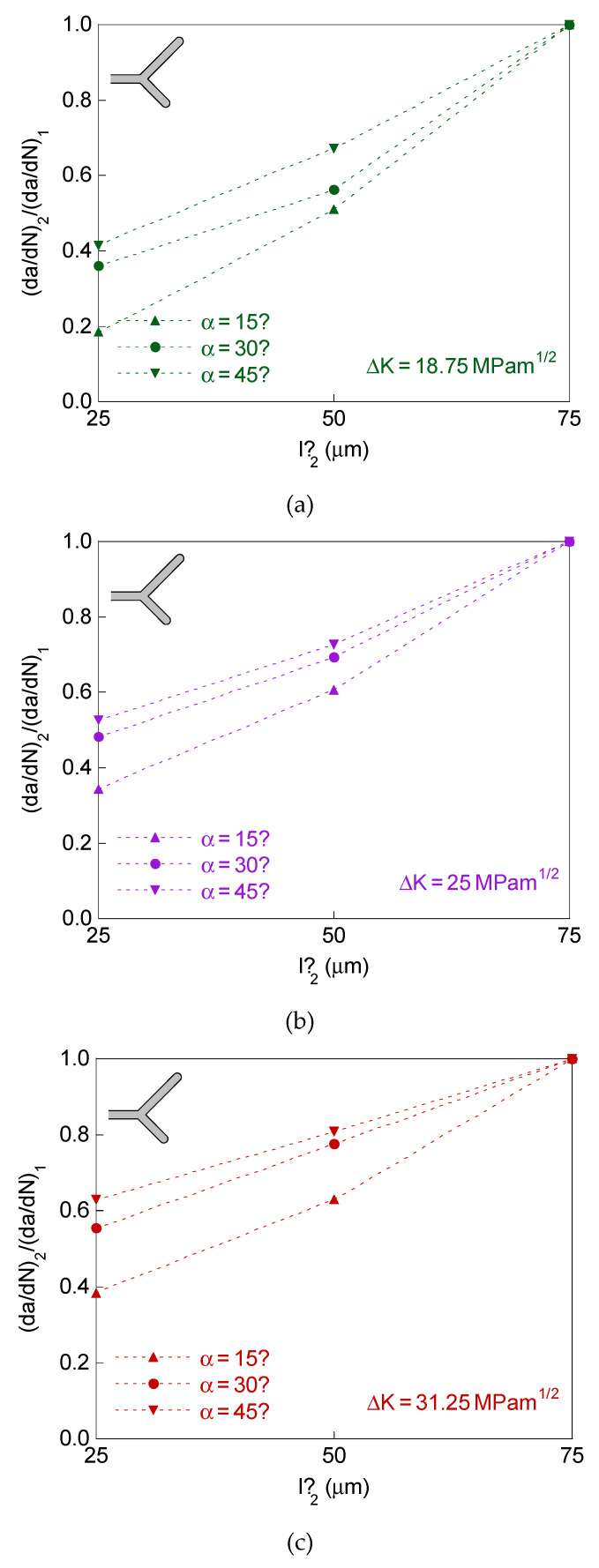
Crack tip growth rates ratio for different initial projected length and equal initial angle in the two branches: (**a**) Δ*K* = 18.75 MPam^1/2^; (**b**) Δ*K* = 25 MPam^1/2^; (**c**) Δ*K* = 31.25 MPam^1/2^.

For the case of a bifurcated crack tip with two branches of different initial projected length and equal initial angle, the defined retardation factor (d*a*/d*N*)_1_/(d*a*/d*N*)_0_ is represented in Figure 4, whereas the crack tip growth rates ratio (d*a*/d*N*)_2_/(d*a*/d*N*)_1_ is plotted in Figure 5.

In this case study there are two different retardation factors associated with the two branches of the bifurcation (since the two crack ends have different propagation rate), the greatest value of the retardation factor appearing in the long-branch, and therefore it is (d*a*/d*N*)_1_/(d*a*/d*N*)_0_ > (d*a*/d*N*)_2_/(d*a*/d*N*)_0_.

The plots in Figure 4 and Figure 5 correspond for the dimensions of the bifurcated crack tip shown in the Table 1, the SIF ranges Δ*K* = 18.75, 25, and 31.25 MPam^1/2^ and *R*-ratio equal to 0. For comparative reasons, the results for the symmetric case of initial projected branch length *l*’ = 75 μm are also represented in these figures.

The results show that the retardation effect increases (i.e., the previously defined retardation factor (d*a*/d*N*)_1_/(d*a*/d*N*)_0_ decreases) with the initial projected short-branch length and with the initial branch angle, and such a retardation effect decreases with the SIF range.

In the matter of the difference between the growth rate at the two crack ends, it increases (i.e., the crack tip growth rates ratio (d*a*/d*N*)_2_/(d*a*/d*N*)_1_ decreases) with the drop in the initial projected short-branch length and in the initial branch angle, not observing a clear trend with the intensity of fatigue (SIF range).

**Figure 6:**
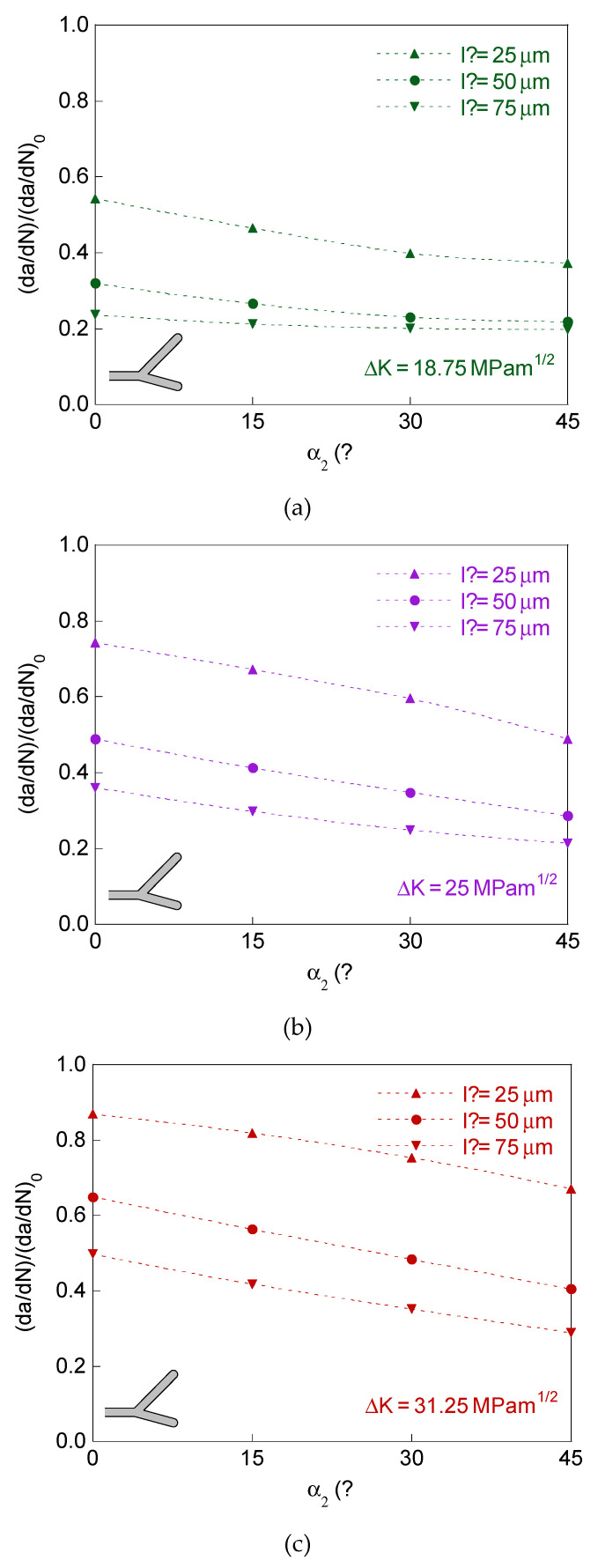
Retardation factor for bifurcated crack tip with equal initial projected length and different initial angle in the two branches: (**a**) Δ*K* = 18.75 MPam^1/2^; (**b**) Δ*K* = 25 MPam^1/2^; (**c**) Δ*K* = 31.25 MPam^1/2^.

**Figure 7:**
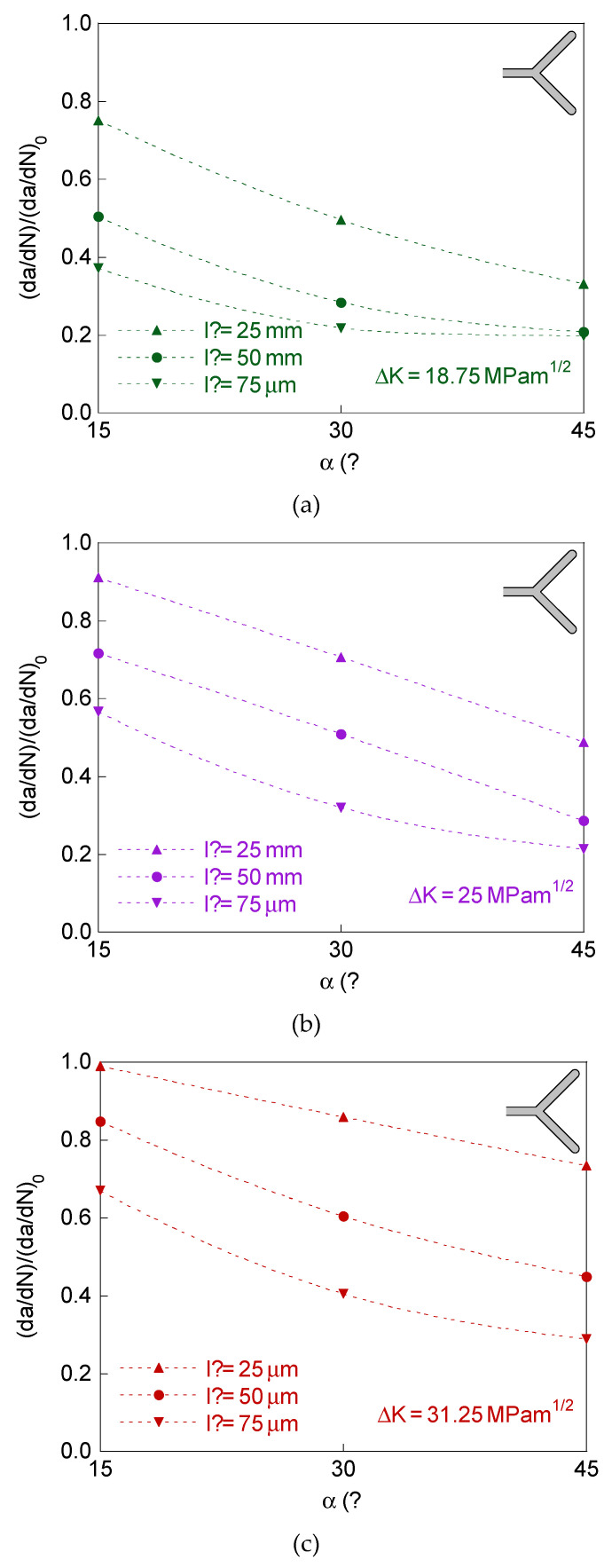
Retardation factor for the symmetric case (equal initial projected length and equal initial angle in the two branches): (**a**) Δ*K* = 18.75 MPam^1/2^; (**b**) Δ*K* = 25 MPam^1/2^; (**c**) Δ*K* = 31.25 MPam^1/2^.

In Figure 6, the retardation factor (d*a*/d*N*)/(d*a*/d*N*)_0_ is represented for bifurcated crack tip with two branches of equal initial projected length and different initial angle, and in Figure 7 for symmetric bifurcated crack tip (equal initial projected length and equal initial angle for the two branches).

For these two cases of equal initial projected branch length, with equal or different initial branch angle, the retardation in the propagation rate is the same for the two crack tips (d*a*/d*N*)/(d*a*/d*N*)_0_ = (d*a*/d*N*)_1_/(d*a*/d*N*)_0_ = (d*a*/d*N*)_2_/(d*a*/d*N*)_0_, despite the asymmetry for the case of different initial branch angle.

The plots in Figure 6 and Figure 7 correspond for the dimensions of the bifurcated crack tip shown in the Table 1, the SIF ranges Δ*K* = 18.75, 25, and 31.25 MPam^1/2^, and *R*-ratio equal to 0. For comparative reasons, the results for the symmetric case of initial branch angle *α* = 45° are also represented in Figure 6.

The results for bifurcated crack tip with two branches of equal initial projected length and different initial angle and for the symmetric case show that the retardation effect increases (i.e., the retardation factor (d*a*/d*N*)/(d*a*/d*N*)_0_ decreases) with the initial projected branch length and with the initial branch angle, decreasing with the SIF range (as in the case of different initial projected branch length).

## 4. Discussion

### 4.1. Different Initial Projected Branch Length

**Figure 8:**
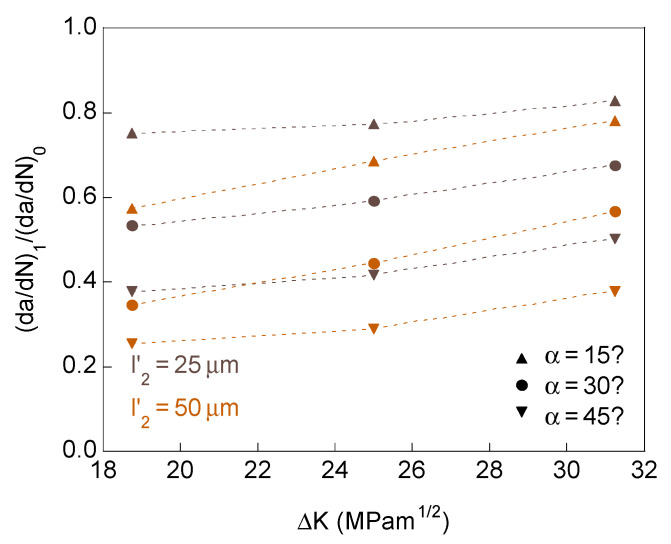
Retardation factor at the long-branch tip as a function of the SIF range for bifurcated crack tip with different initial projected length in the two branches.

For crack bifurcation with two branches of different initial projected length and equal initial angle, the influence of the SIF range on the retardation effect in the long-branch is shown in Figure 8. The retardation factor (d*a*/d*N*)_1_/(d*a*/d*N*)_0_ increases with the SIF range, therefore decreasing the retardation effect with the fatigue intensity.

**Figure 9:**
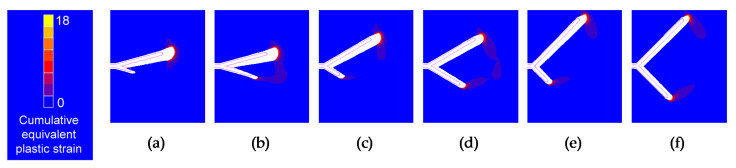
Initial and final crack tip profile and cumulative equivalent plastic strain in the vicinity of bifurcated crack tip with different initial projected branch length and Δ*K* = 31.25 MPam^1/2^: (**a**) *α* = 15°, *l*’_2_ = 25 μm; (**b**) *α* = 15°, *l*’_2_ = 50 μm; (**c**) *α* = 30°, *l*’_2_ = 25 μm; (**d**) *α* = 30°, *l*’_2_ = 50 μm; (**e**) *α* = 45°, *l*’_2_ = 25 μm; (**f**) *α* = 45°, *l*’_2_ = 50 μm.

Increasing the initial projected short-branch length and the initial branch angle have two opposite effects to raise the SIF range. High SIF ranges, low initial projected short-branch lengths, and low initial branch angles make the behavior of the two crack ends approach that of a single end of irregular geometry, tending to propagate towards a more symmetrical form. Low SIF ranges, high initial projected short-branch lengths, and high initial branch angles allow the two crack ends to act more independently. These can be inferred from Figure 9 showing the initial and final crack tip profile, and the cumulative equivalent plastic strain distributions in the vicinity of bifurcated crack tip with distinct initial projected branch length and equal initial branch angle (Δ*K* = 31.25 MPam^1/2^). The cumulative equivalent plastic strain can be considered as a very relevant mechanical parameter characterizing the damage accumulation by cyclic loading [22].

### 4.2. Equal Initial Projected Branch Length

**Figure 10:**
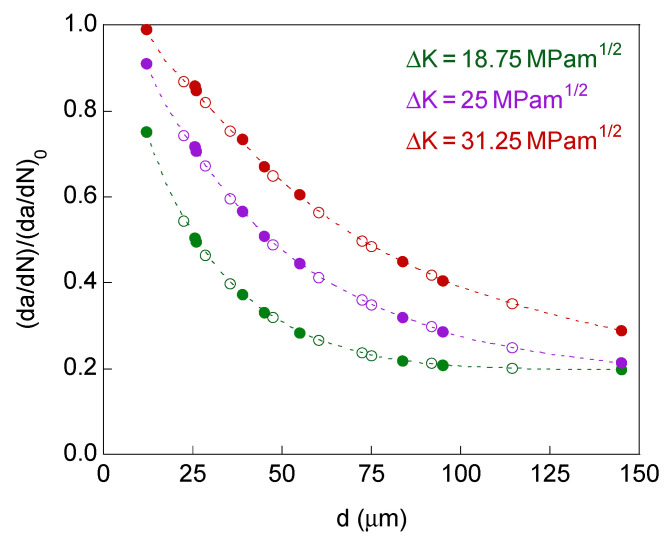
Retardation factor as a function of the distance between crack tips for bifurcated crack tip with the equal initial projected length for the two branches.

If the two crack ends have equal initial projected branch length, regardless of whether the initial angle is the same or different, the retardation effect depends on the initial distance between the two crack tips *d* (Figure 2b,c). For a given SIF range, increasing the initial crack tips distance raises the retardation effect, thus decreasing the crack propagation rate (Figure 10, where the fill marks correspond to identical initial branch angle and the empty marks correspond to different initial branch angle). In relation to the fatigue intensity, the increase in the SIF range decreases the retardation effect (for the same initial crack tips distance). For high fatigue intensities and short initial crack tips distances, it appears retardation factors are very close to one and for low fatigue intensities and long initial crack tips distances the retardation factor seems to reach an asymptotic value close to 0.2.

**Figure 11:**
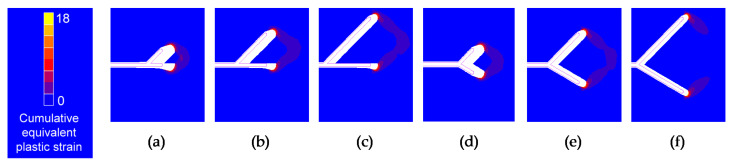
Initial and final crack tip profile and cumulative equivalent plastic strain in the vicinity of bifurcated crack tip with different initial branch angle and Δ*K* = 31.25 MPam^1/2^: (**a**) *α*_2_ = 0°, *l*’ = 25 μm; (**b**) *α*_2_ = 0°, *l*’ = 50 μm; (**c**) *α*_2_ = 0°, *l*’ = 75 μm; (**d**) *α*_2_ = 30°, *l*’ = 25 μm; (**e**) *α*_2_ = 30°, *l*’ = 50 μm; (**f**) *α*_2_ = 30°, *l*’ = 75 μm.

**Figure 12:**
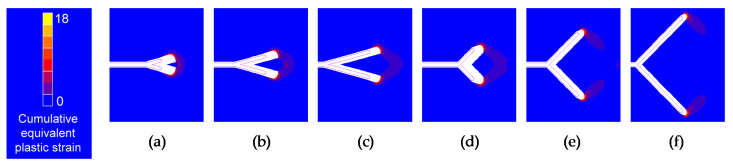
Initial and final crack tip profile and cumulative equivalent plastic strain in the symmetric case with Δ*K* = 31.25 MPam^1/2^: (**a**) *α* = 15°, *l*’ = 25 μm; (**b**) *α* = 15°, *l*’ = 50 μm; (**c**) *α* = 15°, *l*’ = 75 μm; (**d**) *α* = 45°, *l*’ = 25 μm; (**e**) *α* = 45°, *l*’ = 50 μm; (**f**) *α* = 45°, *l*’ = 75 μm.

Initial and final crack tip profile and cumulative equivalent plastic strain for several of the geometries studied when the two tips have identical initial projected branch length can be observed in Figure 11 and Figure 12 (for different initial branch angle and for equal initial branch angle, respectively).

The deformed geometry of the crack shows that for small initial crack tips distance and high SIF range, the envelope of the two crack ends acts as if it were a single tip, while for very high initial crack tips distance and small SIF range, the contours corresponding to the cumulative equivalent plastic strain of the two crack ends are separated, observing little interaction between the two crack tips.

For cases where the retardation effect is almost the same, the aspect of the cumulative equivalent plastic strain around the crack end looks very similar, but smaller in size as the SIF range is smaller, the same as in the case of a crack with the straight tip. In relation to crack opening displacement (*COD*), this seems to be influenced by the initial branch angle, by the initial projected branch length and by the intensity of the fatigue. In addition, if the initial angle of the two branches is different, the *COD* corresponding to the two branches will also be distinct.

## 5. Conclusions

The following scientific conclusions may be drawn with regard to the effect produced by the micro-bifurcation of the crack tip on the plasticity-induced fatigue crack growth rate:

(i) Cracks with bifurcated tip in a plate subjected to remote tensile loading exhibit plastic crack advance in mode I (they show a trend to growth in the direction of the original crack), with retardation in the fatigue crack growth when compared with a fully straight crack (with no branching).
(ii) In the case of asymmetric bifurcation with different initial projected branch lengths, the crack propagation rate is greater at the crack tip corresponding to the long-branch and the retardation effect increases with the initial branch angle and the initial projected branch length of the short-branch and decreases with the SIF range.
(iii) When the initial projected length of the two branches is the same (case symmetric and asymmetric bifurcation with different initial branch angle) the crack propagation rate is equal at the two crack tip. For these cases, the retardation effect increases with the initial distance between the ends of the crack and decreases with the SIF range.

## Figures and Tables

**Table 1:** Initial dimensions of the bifurcated crack tip.

Configuration		Branch Projected Length	Branch Angle
Different projected length and equal angle in the two branches	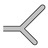	*l*’_1_ = 75 μm *l*’_2_ = 25 and 50 μm	*α* = 15, 30 and 45°
Equal projected length and different angle in the two branches	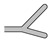	*l*’ = 25, 50 and 75 μm	*α*_1_ = 45° *α*_2_ = 0, 15 and 30°
Symmetric case, equal projected length and angle in the two branches	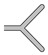	*l*’ = 25, 50 and 75 μm	*α* = 15, 30 and 45°

## Data Availability

Not applicable.

## References

[1] Suresh S. (1983). Micromechanisms of fatigue crack growth retardation following overloads. Eng. Fract. Mech..

[2] Meggiolaro M.A., Miranda A.C.O., Castro J.T.P., Martha L.F. (2005). Stress intensity factor equations for branched crack growth. Eng. Fract. Mech..

[3] Bui T.P., Miyashita Y., Morikage Y., Tagawa T., Handa T., Mutoh Y., Otsuka Y. (2020). Contributions of grain size and crystal orientation to fatigue crack deflection and branching behavior in low carbon steel plates. ISIJ Int..

[4] Toribio J., González B., Matos J.-C. (2015). Analysis of fatigue crack paths in cold drawn pearlitic steel. Materials.

[5] Toribio J., González B., Matos J.-C. (2017). Initiation and propagation of fatigue cracks in cold-drawn pearlitic steel wires. Theor. Appl. Fract. Mech..

[6] Pärletun L.G. (1979). Determination of the growth of branched cracks by numerical methods. Eng. Fract. Mech..

[7] Meggiolaro M.A., Miranda A.C.O., Castro J.T.P., Martha L.F. (2005). Crack retardation equations for the propagation of branched fatigue cracks. Int. J. Fatigue.

[8] Kitagawa H., Yuuki R., Ohira T. (1975). Crack-morphological aspects in fracture mechanics. Eng. Fract. Mech..

[9] Kitagawa H., Yuuki R., Taplin D.M.R. (1978). Analysis of branched cracks under biaxial stresses. Advances in Research on the Strength and Fracture of Materials.

[10] Toribio J., Kharin V. (2013). Simulations of fatigue crack growth by blunting-re-sharpening: Plasticity induced crack closure vs. alternative controlling variables. Int. J. Fatigue.

[11] Toribio J., Kharin V. (2009). Finite-deformation analysis of the crack-tip fields under cyclic loading. Int. J. Solids Struct..

[12] Toribio J., Kharin V. (2007). Large crack tip deformations and plastic crack advance during fatigue. Mater. Lett..

[13] McClung R.C., Thacker B.H., Roy S. (1991). Finite element visualization of fatigue crack closure in plane stress and plane strain. Int. J. Fract..

[14] Tvergaard V. (2004). On fatigue crack growth in ductile materials by crack-tip blunting. J. Mech. Phys. Solids.

[15] Toribio J., Kharin V., Ayaso F.J., González B., Matos J.C., Vergara D., Lorenzo M. (2011). Numerical and experimental analyses of the plasticity-induced fatigue crack growth in high-strength steels. Constr. Build. Mater..

[16] Toribio J., Matos J.C., González B. (2021). Numerical modeling of plasticity-induced fatigue crack growth retardation due to deflection in the near-tip area. Metals.

[17] Proudhon H., Li J., Wang F., Roos A., Chiaruttini V., Forest S. (2016). 3D simulation of short fatigue crack propagation by finite element crystal plasticity and remeshing. Int. J. Fatigue.

[18] Sadananda K., Babu M.N., Vasudevan A.K. (2019). The unified approach to subcritical crack growth and fracture. Eng. Fract. Mech..

[19] Borges M.F., Neto D.M., Antunes F.V. (2020). Revisiting classical issues of fatigue crack growth using a non-linear approach. Materials.

[20] Ortiz González J.A., de Castro J.T.P., Meggiolaro M.A., Gómez Gonzáles G.L., de França Freire J.L. (2020). Challenging the “Δ*K*_eff_ is the driving force for fatigue crack growth” hypothesis. Int. J. Fatigue.

[21] Kujawski D. (2020). Discussion and comments on *K*_OP_ and Δ*K*_eff_. Materials.

[22] Suresh S. (1994). Fatigue of Materials.

